# Preparation of noninfectious scRNAseq samples from SARS-CoV-2-infected epithelial cells

**DOI:** 10.1371/journal.pone.0281898

**Published:** 2023-02-24

**Authors:** Raven M. Osborn, Justin Leach, Michelle Zanche, John M. Ashton, ChinYi Chu, Juilee Thakar, Stephen Dewhurst, Sonia Rosenberger, Martin Pavelka, Gloria S. Pryhuber, Thomas J. Mariani, Christopher S. Anderson

**Affiliations:** 1 Translational Biomedical Sciences Program, University of Rochester School of Medicine and Dentistry, Rochester, New York, United States of America; 2 Clinical and Translational Sciences Institute, University of Rochester School of Medicine and Dentistry, Rochester, New York, United States of America; 3 Department of Microbiology and Immunology, University of Rochester School of Medicine and Dentistry, Rochester, New York, United States of America; 4 Genomics Research Center, Center for Advanced Research Technologies, University of Rochester School of Medicine and Dentistry, Rochester, New York, United States of America; 5 Department of Pediatrics and Center for Children’s Health Research, University of Rochester School of Medicine and Dentistry, Rochester, New York, United States of America; 6 Biophysics, Structural, and Computational Biology Program, University of Rochester School of Medicine and Dentistry, Rochester, New York, United States of America; 7 Department of Biostatistics and Computational Biology, University of Rochester School of Medicine and Dentistry, Rochester, New York, United States of America; 8 Department of Biomedical Genetics, University of Rochester School of Medicine and Dentistry, Rochester, New York, United States of America; 9 Department of Environmental Health and Safety, University of Rochester, Rochester, New York, United States of America; 10 Biosafety Level 3 Facility, Center for Advanced Research Technologies, University of Rochester School of Medicine and Dentistry, Rochester, New York, United States of America; 11 Department of Environmental Medicine, University of Rochester School of Medicine and Dentistry, Rochester, New York, United States of America; 12 Division of Neonatology, Department of Pediatrics, University of Rochester School of Medicine and Dentistry, Rochester, New York, United States of America; Waseda University: Waseda Daigaku, JAPAN

## Abstract

Coronavirus disease (COVID-19) is an infectious disease caused by the SARS coronavirus 2 (SARS-CoV-2) virus. Direct assessment, detection, and quantitative analysis using high throughput methods like single-cell RNA sequencing (scRNAseq) is imperative to understanding the host response to SARS-CoV-2. One barrier to studying SARS-CoV-2 in the laboratory setting is the requirement to process virus-infected cell cultures, and potentially infectious materials derived therefrom, under Biosafety Level 3 (BSL-3) containment. However, there are only 190 BSL3 laboratory facilities registered with the U.S. Federal Select Agent Program, as of 2020, and only a subset of these are outfitted with the equipment needed to perform high-throughput molecular assays. Here, we describe a method for preparing non-hazardous RNA samples from SARS-CoV-2 infected cells, that enables scRNAseq analyses to be conducted safely in a BSL2 facility–thereby making molecular assays of SARS-CoV-2 cells accessible to a much larger community of researchers. Briefly, we infected African green monkey kidney epithelial cells (Vero-E6) with SARS-CoV-2 for 96 hours, trypsin-dissociated the cells, and inactivated them with methanol-acetone in a single-cell suspension. Fixed cells were tested for the presence of infectious SARS-CoV-2 virions using the Tissue Culture Infectious Dose Assay (TCID50), and also tested for viability using flow cytometry. We then tested the dissociation and methanol-acetone inactivation method on primary human lung epithelial cells that had been differentiated on an air-liquid interface. Finally, we performed scRNAseq quality control analysis on the resulting cell populations to evaluate the effects of our virus inactivation and sample preparation protocol on the quality of the cDNA produced. We found that methanol-acetone inactivated SARS-CoV-2, fixed the lung epithelial cells, and could be used to obtain noninfectious, high-quality cDNA libraries. This methodology makes investigating SARS-CoV-2, and related high-containment RNA viruses at a single-cell level more accessible to an expanded community of researchers.

## Introduction

SARS-CoV-2 is a highly infectious RNA virus [1]. Most people infected with the SARS-CoV-2 will experience mild to moderate respiratory illness and recover without requiring additional treatment. However, many become severely ill and require medical intervention. Previous studies have shown that a robust innate immune response to viral respiratory pathogens is correlated with less severe outcomes, including a reduced risk of death in humans and non-human primates [2,3]. As a result, there is considerable interest in analyzing the host response to SARS-CoV-2 on a molecular level.

Host responses to viral infection are typically informed by the analysis of tissue samples from infected subjects, as well as studies from tractable experimental systems such as cell culture models that permit the careful dissection of variables such as time after infection, viral dose, viral strain, etc. In recent years, data collection for the characterization and quantification of biological mechanisms and host responses to viral infections has been conducted and analyzed on a per cell basis using methods such as scRNAseq [4–7]. In the case of SARS-CoV-2, such studies are complicated by the fact that the infectious virus must be propagated under BSL3 containment conditions.

Unfortunately, high containment biologic laboratories such as BSL3 facilities are rare. In the United States, only 190 BSL3 laboratories were registered with the Federal Select Agent Program (FSAP), according to the FSAP’s most recent annual report [8]. While this is likely an underestimate of the total number of BSL3 labs in the U.S. capable of working with SARS-CoV-2, since the virus is not registered as a federal select agent, it is true that only a small subset of U.S. BSL3 labs are outfitted with the equipment needed to perform high-throughput molecular assays. As a result, the ability to perform in-depth experimental analyses of the host cell response to SARS-CoV-2 is significantly constrained–impeding national and global efforts to understand and treat the COVID-19 pandemic.

Here, we describe a method for preparing non-hazardous RNA samples from SARS-CoV-2 infected cells, that enables subsequent single-cell RNA sequencing (scRNAseq) analyses to be conducted safely in a BSL2 facility. The methods described are expected to make molecular assays of SARS-CoV-2 cells accessible to a greatly expanded community of researchers and may also be applicable to other, related, high-containment RNA viruses.

## Materials and methods

The protocol described in this peer-reviewed article is published on protocols.io (https://dx.doi.org/10.17504/protocols.io.kqdg3999eg25/v1) and is included for printing purposes as S1 File.

### Primary human cells

Donor lungs were provided through the federal United Network of Organ Sharing via the National Disease Research Interchange and the International Institute for Advancement of Medicine. With written consent, dissociated lung cells from deceased donors were entered into the LungMAP program’s biorepository and were utilized in this study. The University of Rochester’s Institutional Review Board approved and oversaw this study (RSRB00047606).

### Viruses

The following reagents were deposited by the Centers for Disease Control and Prevention and obtained through BEI Resources, NIAID, NIH: SARS-Related Coronavirus 2, Isolate USA-WA1/2020, NR-52281 and SARS-Related Coronavirus 2, Isolate Hong Kong/VM20001061/2020, NR-52282. SARS-CoV-2 was propagated and titered using African green monkey kidney epithelial Vero E6 cells (American Type Culture Collection, CRL-1586) in Eagle’s Minimum Essential Medium (Lonza, 12-125Q) supplemented with 2% fetal bovine serum (FBS) (Atlanta Biologicals), 2 mM l-glutamine (Lonza, BE17-605E), and 1% penicillin (100 U/ml) and streptomycin (100 ug/ml). Virus stock was stored at − 80°C. All work involving infectious SARS-CoV-2 was performed in the Biosafety Level 3 (BSL-3) core facility of the University of Rochester, with institutional biosafety committee (IBC) oversight.

### SARS-CoV-2 infections of kidney epithelial cells

Vero E6 cells were plated at approx. 4.3e+4/cm^2^ and left to rest for 18–24 hours. When 80% confluent, they were washed with PBS and inoculated with SARS-CoV-2 (Hong Kong/VM20001061/2020) at a multiplicity of infection (MOI) of 0.1 in infection medium (Eagle’s Minimum Essential Medium (Lonza, 12-125Q) supplemented with 2% fetal bovine serum (FBS) (Atlanta Biologicals), 2 mM l-glutamine (Lonza, BE17-605E), and 1% penicillin (100 U/ml) and streptomycin (100 μg/ml)).

### Tissue culture infectious dose assay

Viral titers were determined using the tissue culture infectious dose (TCID) assay on triplicate wells of an 80% confluent monolayer of Vero E6 cells in a 96-well microtiter plate format using a 1:3 dilution factor; virus infection was assessed following 4 days of incubation at 37°C in a CO_2_ incubator by microscopic examination of cytopathic effects (CPE). The infectious dose (log10 TCID50/ml) was calculated using the Spearman-Kärber method [9,10].

### Cell culture on air-liquid interface

Primary human lung cells were cultured on an air-liquid interface as previously described [11,12]. Briefly, lung tissue issues were digested with a protease cocktail and cells were then cultured on a collagen-coated transwell plate (Corning, 3470) until each well reached a transepithelial electrical resistance (TEER) measurement of >300 ohms. Cells were then placed at air-liquid interface (ALI) by removing media from the apical layer of the transwell chamber and continuing to feed cells on the basolateral layer as they differentiate. Cells were differentiated for 4–5 weeks at ALI before use in experiments.

### SARS-CoV-2 infections of airway epithelial cells

The apical layer of primary lung cells that had been cultured at the air-liquid interface for about 4–5 weeks were inoculated with SARS-CoV-2 (BEI, NR-52281, hCoV-19/USA-WA1/2020) at a MOI of 5 (titered in VeroE6 cells) in phosphate-buffered saline containing calcium and magnesium (PBS++; Gibco, 14040–133) and incubated at 37°C for 1.5 hours. The infectious solution was then removed and the apical layer washed with PBS++. Cells were then incubated for 48 hours.

### SARS-CoV-2 inactivation and scRNA-seq sample preparation

Cultured cells were washed by dispensing and aspirating 37°C HEPES buffered saline solution (Lonza, CC-5022), and then dissociated with 0.025% Trypsin/EDTA (Lonza,CC-5012) for 10 min at 37°C. Dissociated cells were aspirated using a wide-bore pipette tip and placed in a tube containing ice-cold Trypsin Neutralization Solution (Lonza, CC5002); this was repeated to maximize cell collection. Cells were then pelleted by centrifugation, resuspended in chilled HEPES, and centrifugally pelleted once more. Using a wide-bore pipette tip, the supernatant was then removed and the cell pellet was resuspended in 100ul chilled 1X DPBS. Next, 1ml of a chilled 1:1 methanol acetone mixture was added to the cells in a dropwise manner with continuous gentle agitation. Cells were incubated on ice for 1 hour, washed in PBS++, counted, and finally resuspended in cold SSC cocktail (3× Lonza AccuGENE SSC, BMA51205 + 0.04% BSA + 1mM DTT + 0.2 U/ul RNase1 inhibitor).

### Flow cytometric analysis of cell viability

Uninfected VERO E6 cells were subjected to the full inactivation protocol (S1). Cells were then stained with 10uM of calcein green (Invitrogen, C3100MP) and incubated for 20 minutes at room temperature. Flow cytometry data were then collected and analyzed using instrument BD Accuri C6 and FCSexpress 7 software (Version 7.12.0007, respectively).

### Library preparation and sequence mapping

Following SARS-CoV-2 inactivation and rehydration, cell suspensions were processed to generate single-cell RNA-Seq libraries using Chromium Next GEM Single Cell 3′ GEM, Library and Gel Bead Kit v3.1 (10x Genomics), per the manufacturer’s recommendations, as summarized below. To minimize the addition of the rehydration buffer, a maximum of 4ul cell suspension was used in the GEM (Gel Bead-in-Emulsion) generation step. Subsequently, samples were loaded on a Chromium Single-Cell Instrument (10x Genomics, Pleasanton, CA, USA) to generate single-cell GEMs. GEM reverse transcription (GEM-RT) was performed to produce a barcoded, full-length cDNA from poly-adenylated mRNA. After incubation, GEMs were broken, the pooled GEM-RT reaction mixtures were recovered, and cDNA was purified with silane magnetic beads (DynaBeads MyOne Silane Beads, PN37002D, ThermoFisher Scientific). The purified cDNA was further amplified by PCR to generate sufficient material for library construction. Enzymatic fragmentation and size selection was used to optimize the cDNA amplicon size and indexed sequencing libraries were constructed by end repair, A-tailing, adaptor ligation, and PCR. Final libraries contain the P5 and P7 priming sites used in Illumina bridge amplification. Sequence data was generated using Illumina’s NovaSeq 6000. Samples were demultiplexed and counted using 10x cellranger version 6.0.1 with standard parameters. Samples were aligned against a combined reference containing the 10x provided human reference (GRCh38-2020-A) and NCBI GenBank reference sequence MT644268.

### scRNA-seq quality control analysis

Pre-processing and quality control analysis of scRNA-seq count data were performed using the Seurat (version 4.1.0) [13] and scDblFinder (version 1.4.0) [14] R packages, using R version 4.0.5. Cells with more than 6000 features and exceeding a mitochondrial percentage of 10% were excluded in the analysis, in line with previous studies [11].

## Results

### Methanol-acetone fixation inactivates SARS-CoV-2 in VeroE6 cells

**Fig 1:**
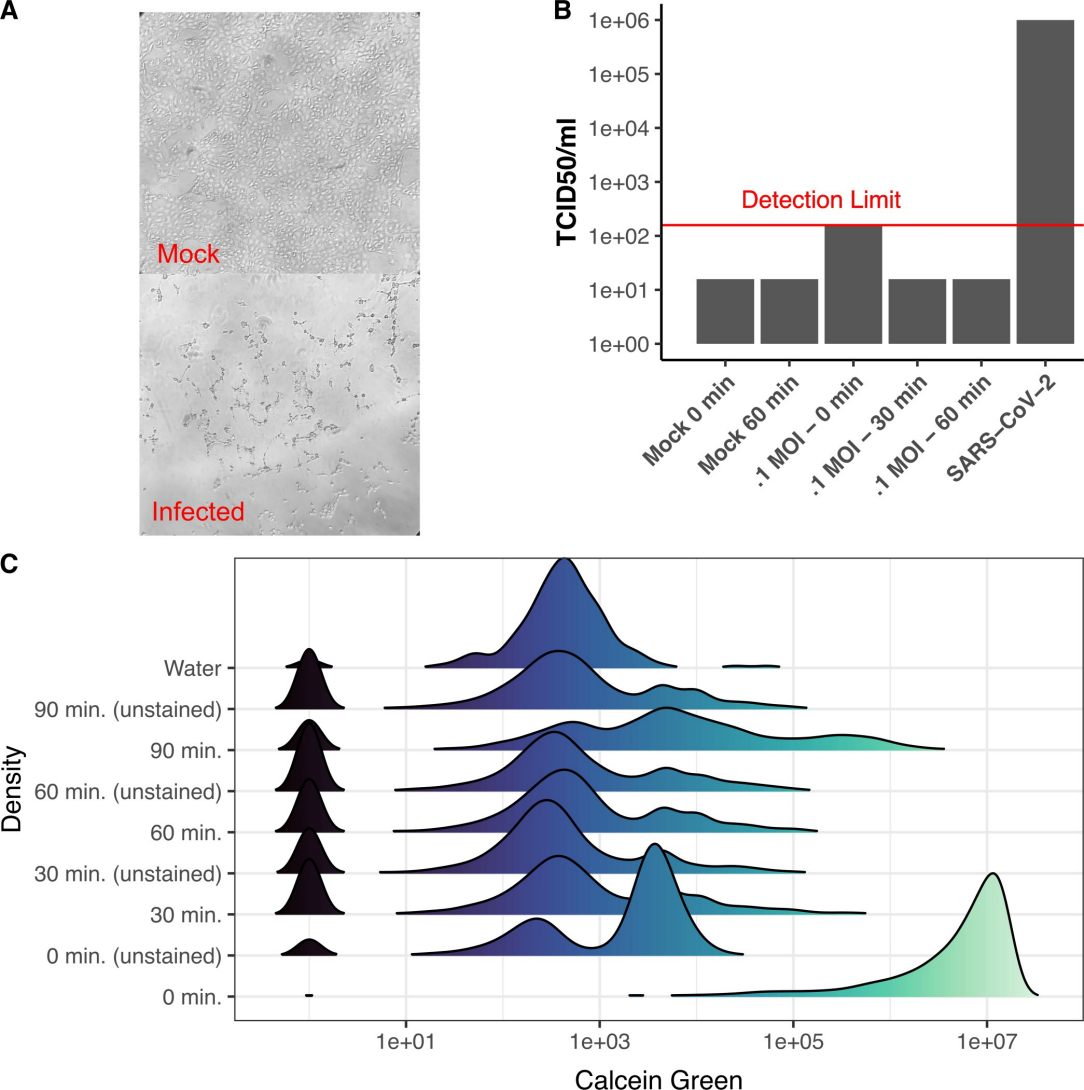
SARS-CoV-2 inactivation and cellular fixation. VeroE6 cells were infected with SARS-CoV-2 for 96 hrs and fixed with methanol-acetone. They were then examined for the presence of SARS-CoV-2-induced cytopathic effects. (A), infectious virus (B) and cell viability (C). (A) Representative images of cytopathic effects of SARS-CoV-2 at 96 hrs post infection at 10X magnification. (B) Bar plot of TCID_50_ results. Time indicates the length of methanol-acetone fixation. (C) Density plot showing the kernel density estimation of flow cytometry results from uninfected cells using the cell viability dye, calcein green. M-A indicates methanol-acetone fixation. *indicates unstained control.

VeroE6 cells were infected with SARS-CoV-2 and imaged at 96 hours to confirm viral infection, as assessed by cytopathic effects (Fig 1A). The cells were then fixed with methanol-acetone for 30 or 60 minutes, and tested for the presence of residual, infectious SARS-CoV-2 by TCID_50_ analysis (Fig 1B). No infectious virus was detected in samples that had been incubated with methanol-acetone for either 30 or 60 minutes (Fig 1B). Virus from infected cells that were not incubated with methanol-acetone was detected at a low concentration, nearly a 4-log reduction compared to the positive control. Furthermore, uninfected cells that had been fixed with methanol-acetone for 30, 60, or 90 minutes were found to be non-viable, as assessed by staining with calcein green (Fig 1C).

### Methanol-acetone fixation of SARS-CoV-2 infected cells is compatible with the isolation of high quality/integrity RNA

**Fig 2:**
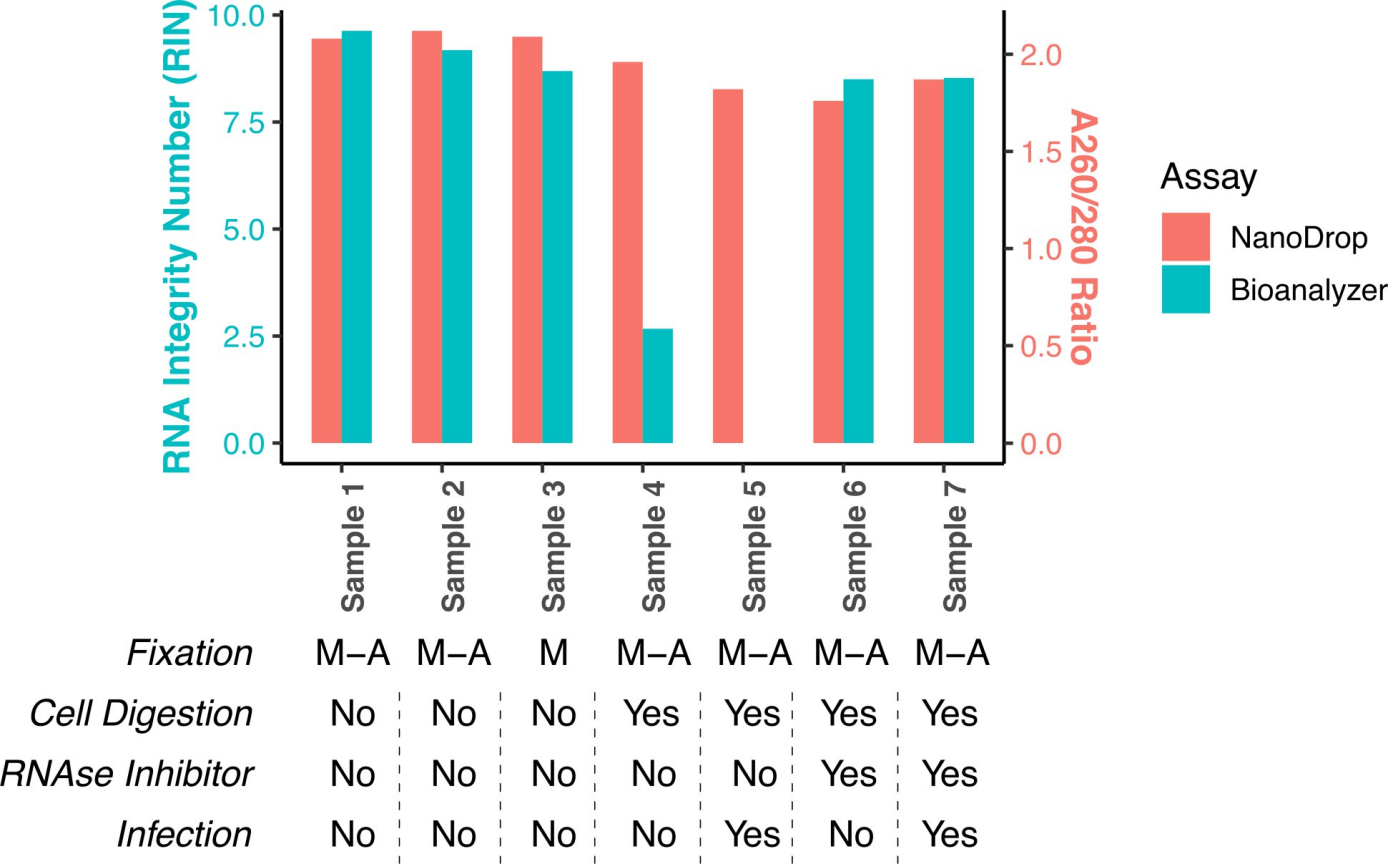
RNA integrity and purity assessment by NanoDrop and Bioanalyzer. Cultures of primary human lung epithelial cells were differentiated on an air-liquid interface, and in some cases infected with SARS-CoV-2 (denoted by the label “infection”). Cells were then removed from transwells either by trypsinization (denoted by the label “digestion”) or using cold saline alone. Cells were then fixed with methanol-acetone (M-A) or methanol alone (M), and RNA was extracted, either in the absence or presence of an RNAse 1 inhibitor (as indicated by the pertaining label). RNA quality/integrity was then measured. Results denote RNA integrity number (RIN), as measured using a Bioanalyzer (left axis, green bars) or A_260_/A_280_ ratio, as measured using a NanoDrop instrument (right axis, orange bars).

We evaluated the impact of various steps in our cell inactivation protocol on the quality/integrity of RNA isolated from the final, fixed cell preparations. To do this, we isolated RNA from cells that had been exposed to: (i) SARS-CoV-2 (“infection”); (ii) trypsin digestion (“digestion”) vs. physical dissociation, (iii) fixation with methanol-acetone (“M-A”) vs. methanol alone (“M”), and (iv) isolation of RNA in the absence or presence of an RNAse 1 inhibitor (“RNAse 1 inhibitor”), as illustrated in Fig 2. RNA integrity and quality was assessed by (i) measuring RNA integrity number (RIN), using a Bioanalyzer (left axis, green bars) or A_260_/A_280_ ratio, and (ii) measuring RNA quality, using a NanoDrop instrument (Fig 2).

As shown in Fig 2, all sample preparations resulted in a Nanodrop A_260_/A_280_ measurement between 1.76–2.12, indicating that the RNA had low contaminants. In parallel, we also measured RNA integrity number (RIN) using a Bioanalyzer which is typically used as a measure of RNA library quality during scRNAseq. Infection status or fixation using either methanol-acetone and or methanol alone did not reduce RNA integrity, with samples measuring 8.69 and 9.63, respectively. Cellular digestion with trypsin, however, greatly reduced RNA integrity and yielded RIN values below 3. However, the addition of RNase1 inhibitor to the sample preparation of digested cells returned the RIN to around 8.52 (Fig 2).

### Methanol-acetone fixation of SARS-CoV-2 infected cells is compatible with the generation of robust scRNAseq data from primary lung epithelial cells

**Fig 3:**
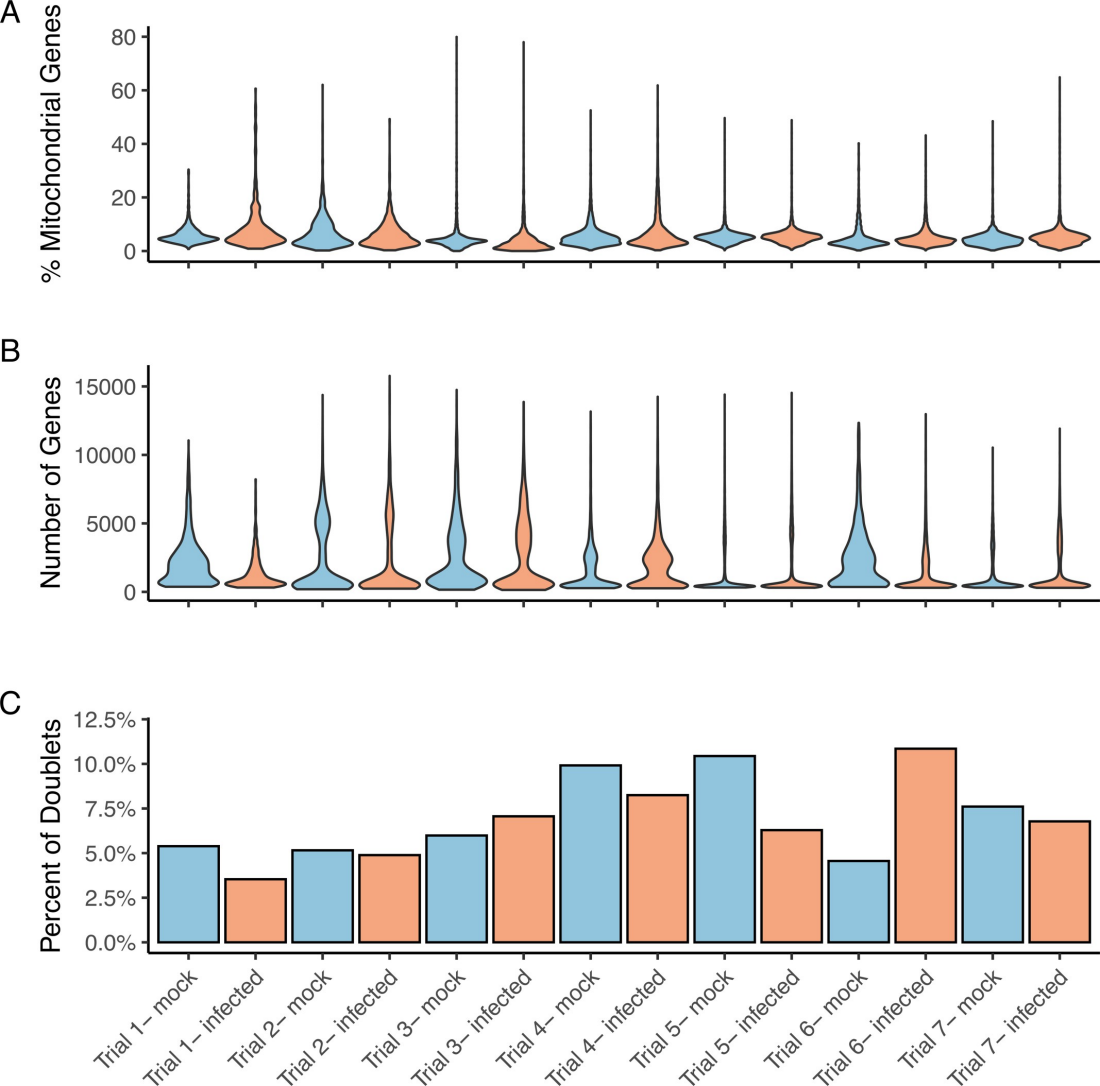
Quality assessment of scRNAseq data from primary lung epithelial cells. Seven independent cultures (“trials”) of primary human lung epithelial cells (each from a different donor) were differentiated on an air-liquid interface, and then infected with SARS-CoV-2 (“infected”; see Methods) or subjected to mock infection (“mock”). Cells were then fixed with methanol-acetone, and RNA isolated in the presence of an RNAse 1 inhibitor. RNA samples were then subjected to scRNAseq analysis, and assessed for common quality control metrics. (A) Violin plot depicting the percentage of mitochondrial genes in each cell. (B)Violin plot of log_10_ normalized detected reads per cell. (C) Bar plot of doublet proportion for each sample.

Seven independent cultures (“trials”) of primary human lung epithelial cells (each from a different donor) were differentiated on an air-liquid interface, and then infected with SARS-CoV-2 (“infected”) or subjected to mock infection (“mock”) (Fig 3). Single cells were then dissociated, fixed with methanol-acetone, and RNA isolated in the presence of an RNAse 1 inhibitor–based on the results presented in Fig 2.

RNA samples were then subjected to scRNAseq analysis, and assessed for common quality control metrics. Using our methodology, we found that 98% of cells had < 20% detected mitochondrial genes and that 92% of cells had < 10% (Fig 3A). The percentage of detected mitochondrial genes was consistent in all trials and there was no difference between infected and uninfected samples (Fig 3A). Similarly, the number of detected genes was consistent in all trials and there was no difference between infected and uninfected samples (Fig 3B). We found that 95% of all cells had less than 6000 detected genes (Fig 3B). Combined, we found that 87% of detected cells had less that 10% mitochondrial genes and less than 6000 genes. Finally, most trials showed a doublet proportion of less than 8%, apart from two samples whose doublet proportion was 10% (Fig 3C). Data in S1 and S2 Datasets.

## Discussion

The objective of this project was to derive a unified workflow to both inactivate SARS-CoV-2 and safely prepare high quality samples for scRNAseq analysis in a BSL2 facility–thereby making molecular assays of SARS-CoV-2 cells accessible to a much larger community of researchers. Our approach was to combine and optimize methods from previous studies for inactivating SARS-CoV-2 or using fixed cells for scRNAseq [15–17]. To this end, we used methanol and acetone in equal volumes to inactivate cell-associated virus from infected VeroE6 cells while preserving cellular and RNA integrity for scRNAseq. Our data show that methanol-acetone fixation significantly reduced the infectious virus titer below the limit of detection, and that it also eliminated cell viability after just 30 minutes. Therefore, our protocol is efficient at fixing and inactivating SARS-CoV-2 in lung epithelial cells.

Poor RNA integrity resulting from suboptimal scRNAseq sample preparation can lead to noisy data and inaccurate analyses [18]. To test if our cellular digestion or fixation protocols had negative effects on the resulting RNA quality, we used primary human lung epithelial cells that had been differentiated on air liquid interface as a biologically relevant model system, since this culture method induces the formation of tight junctions and mimics the pseudostratified phenotype of the airway epithelium *in vitro* [11,19,20]. We extracted and assessed RNA quality at every crucial step of our scRNAseq sample preparation protocol. As reported by others, we found that digestion of cells with trypsin results in reduced RNA integrity and quality, likely due to the presence of ribonucleases [21]. We also found this could be overcome through the addition of an RNAse1 inhibitor in the scRNAseq rehydration buffer. This demonstrates that our sample preparation protocol does not negatively impact RNA integrity.

Ultimately, the most important readout of RNA quality/integrity in our experimental system is that it should be compatible with the generation of reproducible, high-quality single-cell RNA data. Two common quality control measurements to determine if sequencing data should be used in downstream analysis are: (i) the percentage of the detected mitochondrial genes in a cell, and (ii) the presence of cell aggregates. In a typical single-cell quality control workflow, the percentage of mitochondrial genes has been used as an indication of cellular health. The presence of a high percentage of mitochondrial genes is indicative of reduced cell viability (apoptosis or lysis), and can create noise in downstream analysis [22]. Moreover, it has been reported that excessive trypsinization can damage cell membranes and kill cell thereby resulting in poor quality scRNAseq data [23]. Reassuringly, we found that our protocol consistently produced scRNAseq data that fell within the acceptable range for mitochondrial genes as well as the number of detected genes [11,24]. We also examined our sequencing data for the presence of doublets (Fig 3), which can occur as a result of the formation of cellular aggregates (e.g, due to the excessive sample dehydration). This is a concern both in terms of data quality and in terms of microfluidics, since cell aggregates can clog the microfluidics systems of next-generation sequencing platforms and be a source of background noise in the resulting data. To measure the presence of doublets, we quantified the number of detected genes for each instance using scDblFinder, a package that uses a unique reimplementation of the Amulet detection method to find the percentage aggregates in scRNAseq data. An abnormally high number of detected genes could indicate a doublet or multiplet [14]. When comparing our doublet detection rates to other studies, we found that all of our trials were consistent with previous reports using scRNAseq from lung epithelial cells differentiated on an air-liquid interface [11]. Thus, we concluded that our protocol is not associated with a significant increase in apoptotic cells, cell lysis or cell aggregates.

One of the unexpected findings from this study was the variability in the number of detected doublets/multiplets from trial to trial (Fig 3). This variability may be an effect of using pipette tips with different bore radii. While wide-bore pipette tips were used in later trials to reduce the time to dissociate cells in a single-cell suspension, our initial trials used standard-bore pipette tips—which may have contributed to fewer cell aggregates in these experiments. This fixation protocol may be used for other high-throughput single-cell assays such as single-cell proteomics, DNA sequencing, epigenomics, or immunofluorescence protocols. However, users should use methanol-acetone before staining, as methanol and acetone are known to denature proteins. This alcohol-protein interaction could reduce the signals from protein-based fluorophores like PE, APC, and PerCP.

## Conclusion

In conclusion, the overall purpose of this study was to create an accessible protocol to generate non-infectious scRNAseq sample preparations from cells infected with live SARS-CoV-2. The methods described are expected to make molecular assays of SARS-CoV-2 cells accessible to a greatly expanded community of researchers, and may also be applicable to other, related, high-containment RNA viruses.

## Supporting information

S1 File Step-by-step protocol, also available on protocols.io. SARS-CoV-2 inactivation and scRNAseq sample preparation protocol.
(PDF)

S1 Dataset Cell quality data for all trials. Columns contain the cell identifier (cell_ID), the total number of molecules detected within a cell (nCount_RNA), the number of genes detected in each cell (nFeature_RNA), and the percentage of mitochondrial genes detected in each cell (percent.mitochrondrial).
(CSV)

S2 Dataset Doublet information for each sample. Columns contain the capture identifier (Sample), the number of detected single cells in capture (Singlets), the number of detected aggregates in a capture (Doublets), the total number of cell stances in a capture (Total), and the proportion of cell aggregates within a capture (Dbl.Prop).
(CSV)
