# Supplementary material for: Preparation of noninfectious scRNAseq samples from SARS-CoV-2-infected epithelial cells
Source: PLoS One. 2023 Feb 24;18(2):e0281898. doi: 10.1371/journal.pone.0281898 (PMC9956660; doi:10.1371/journal.pone.0281898)
Supplement: S1 File — SARS-CoV-2 inactivation and scRNAseq sample preparation protocol. (PDF) [file pone.0281898.s001.pdf]

SARS-CoV-2 inactivation and scRNAseq sample preparation protocol

Raven M Osborn[1,2,3], Justin Leach[3], Michelle Zanche[4], John M. Ashton[4], ChinYi Chu[5], Juilee Thakar[1,2,3,6,7,8], Stephen Dewhurst[2,3], Sonia Rosenberger[9,10], Martin Pavelka[3,10], Gloria S Pryhuber[5,11], Thomas J. Mariani[5], Christopher Anderson[5,12]

[1]Translational Biomedical Sciences Program, University of Rochester School of Medicine and Dentistry, Rochester, New York, United States of America;
[2]Clinical and Translational Sciences Institute, University of Rochester School of Medicine and Dentistry, Rochester, New York, United States of America;
[3]Department of Microbiology and Immunology, University of Rochester School of Medicine and Dentistry, Rochester, New York, United States of America;
[4]Genomics Research Center, Center for Advanced Research Technologies, University of Rochester School of Medicine and Dentistry, Rochester, New York, United States of America;
[5]Department of Pediatrics and Center for Children's Health Research, University of Rochester School of Medicine and Dentistry, Rochester, New York, United States of America;
[6]Biophysics, Structural, and Computational Biology Program, University of Rochester School of Medicine and Dentistry, Rochester, New York, United States of America;
[7]Department of Biostatistics and Computational Biology, University of Rochester School of Medicine and Dentistry, Rochester, New York, United States of America;
[8]Department of Biomedical Genetics, University of Rochester School of Medicine and Dentistry, Rochester, New York, United States of America;
[9]Department of Environmental Health and Safety, University of Rochester, Rochester, New York, United States of America;
[10]Biosafety Level 3 Facility, Center for Advanced Research Technologies, University of Rochester School of Medicine and Dentistry, Rochester, New York, United States of America;
[11]Department of Environmental Medicine, University of Rochester School of Medicine and Dentistry, Rochester, New York, United States of America;
[12]Division of Neonatology, Department of Pediatrics, University of Rochester School of Medicine and Dentistry, Rochester, New York, United States of America

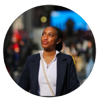

Raven M Osborn
University of Rochester School of Medicine and Dentistry

Protocol Info: Raven M Osborn, Justin Leach, Michelle Zanche, John M. Ashton, ChinYi Chu, Juilee Thakar, Stephen Dewhurst, Sonia Rosenberger, Martin Pavelka, Gloria S Pryhuber, Thomas J. Mariani, Christopher Anderson . SARS-CoV-2 inactivation and scRNAseq sample preparation protocol. **protocols.io** https://protocols.io/view/sars-cov-2-inactivation-and-scrnaseq-sample-prepar-cinyudfw

Created: Nov 01, 2022

Last Modified: Jan 10, 2023

PROTOCOL integer ID: 72120

Keywords: SARS-CoV-2, SARS-Associated coronavirus-2, Severe Acute Respiratory Syndrome Coronavirus 2, COVID-19, Coronavirus Disease, scRNAseq, Single Cell RNA sequencing, BSL3, Biosafety Level 3

ABSTRACT

Coronavirus disease (COVID-19) is an infectious disease caused by the SARS coronavirus 2 (SARS-CoV-2) virus. Direct assessment, detection, and quantitative analysis using high throughput methods like single-cell RNA sequencing (scRNAseq) is imperative to understanding the host response to SARS-CoV-2. One barrier to studying SARS-CoV-2 in the laboratory setting is the requirement to process virus-infected cell cultures, and potentially infectious materials derived therefrom, under Biosafety Level 3 (BSL-3) containment. However, BSL3 laboratory facilities are rare and only a subset of these facilities are outfitted with the equipment needed to perform high-throughput molecular assays. Here, we describe a method for preparing non-hazardous RNA samples from SARS-CoV-2 infected cells, that enables scRNAseq analyses to be conducted safely in a BSL2 facility – thereby making molecular assays of SARS-CoV-2 cells accessible to a much larger community of researchers.

ATTACHMENTS

568-1199.docx

Guidelines:

This procedure must be performed in a biosafety cabinet according to the approval and Standard Operation Procedures for your institution's Biosafety Level 3 program.

This protocol was created in the University of Rochester's Center for Advanced Research Technologies Biosafety Level 3 (BSL3) facility, under the University of Rochester's Institutional Biosafety Committee (IBC) oversight.

Notes:

- This procedure assumes a SARS-CoV-2 – infected cell culture as a starting point.
- No viable virus can be taken out of the BSL3 facility unless inactivated.
- All pipet tips are filter barrier tips. Follow procedures in "Pipetting".
- No vacuum suction is used to remove media or wash buffers from tubes or tissue culture wells; only use pipettors.
- Troughs for multichannel pipettors are only used with media, not virus suspensions. Tips from multichannel pipets are rinsed in a container of disinfectant and then ejected into a biohazard bag in a container. Both containers must be wide enough to fit the pipettor.
- No work is conducted on an open bench, and all cultures are in secondary containers when outside of the biosafety cabinet. The only exception is when culture trays or flasks are examined with the inverted microscope. In these instances, use of a Powered Air Purifying Respirator (PAPR) is required.
- All secondary containers should be labeled with your name, date, and "SARS-CoV-2".

Materials

HEPES Buffered Saline Solution 100 mL **Lonza Catalog #CC-5022**
Trypsin/EDTA 0.025% solution for use in cell culture **Lonza Catalog #CC-5012**
Trypsin Neutralizing Solution for use in cell culture 100 mL **Lonza Catalog #CC-5002**

- 1X DPBS
- methanol
- acetone
- 3× Lonza AccuGENE SSC-0.04% BSA
- 1mM DTT
- RNase Inhibitor
- trypan blue
- Vero E6 cells
- media (EMEM/10% FBS/1% Pen/Strep)
- infection medium (EMEM/3%FBS)
- c-chip hemocytometer
- Vesphene® III disinfectant
- metal petri dish can
- Plastic roller bottle
- Biohazard waste bag
- 500 ml squirt bottle
- markers
- pipetmen
- pipette aid

Lifting cells from tissue culture plate

1 Set up the biosafety cabinet according to your institution's BSL3 biosafety cabinet setup standard operating procedure or the "Basic Biosafety Cabinet Setup" supplied in this protocol.

Note

Note: This procedure was performed on cells in a 24-well transwell plate. Please adjust the reagent quantities according to the surface area of your cell culture plate.

2 Transfer the tissue culture plates, in a secondary container, from the CO_2 incubator to the biosafety cabinet.

3 If supernatant from infected cells is of interest, collect supernatant into screw-capped microcentrifuge tubes and incubate at 65 °C for 01:00:00 to inactivate the virus. 1h

4 Wash cells by dispensing and aspirating HEPES buffered saline solution (Lonza CC-5022).

5 Add 500 µL Trypsin-EDTA (Lonza Catalog #: CC-5012) and incubate for 00:10:00 at 37 °C . 10m

6 Gently pipette up and down to dissociate cells using wide-bore 200-ul pipette tip.

7 Transfer cells to a 1.5-ml tube containing 500 µL of ice-cold Trypsin-Neutralization-Solution (CC-5002).

8 If needed, add an additional 500 µL Trypsin-EDTA to cells and incubate at 37 °C for no longer than 00:30:00 to maximize collection of cells. 30m

9 Transfer cells to the same cold Trypsin-Neutralization-Solution 1.5-ml tube.

10 Centrifuge cells at 300 x g, 00:05:00 according to your institution's BSL3 centrifugation procedures or the "Centrifugation" procedure supplied in this protocol. Remove the supernatant without disrupting the cell pellet. 5m

11 Using a wide-bore pipette tip, add 1 mL chilled HEPES and gently pipette mix 10x or until cells are resuspended.

12 Centrifuge cells at 300 x g, 00:05:00 . 5m

SARS-CoV-2 inactivation with 1:1 methanol-acetone

13

Note

Notes:

- May remove cells from BSL3 facility after final incubation step. Follow your institution’s procedure for BSL3 item removal or the "Standard Item Removal Procedure” supplied.
- For initial tests, validate SARS-CoV-2 inactivation using “Inactivated SARS-Cov-2 TCID Determination” procedure.

Remove the supernatant without disrupting the cell pellet.

14 Using a wide-bore pipette tip, add 100 µL chilled 1X DPBS and gently pipette mix 10x or until cells are resuspended.

15 In a dropwise fashion, add chilled 1 mL 1:1 methanol acetone mixture to cells.

Note

To avoid clumping while adding the methanol-acetone mixture, gently stir the cell suspension with the pipette tip in the microcentrifuge tube.

16 Incubate On ice for 01:00:00 . 1h

Single cell RNAseq preparation

17 5m

Note

Notes:

- All steps must be performed On ice .
- Keep cells On ice until use with scRNAseq procedure (ideally less than 01:00:00).
- Can be performed in BSL2 facility.
- May remove cells from BSL3 facility after final incubation step. Follow your institution's procedure for BSL3 item removal or the "Standard Item Removal Procedure" supplied.

Centrifuge cells at 1000 x g, 4°C, 00:05:00 .

18 Remove the methanol-acetone mixture without disrupting the cell pellet.

19 Resuspend cells in 500 µL of ice-cold SSC cocktail (3× Lonza AccuGENE SSC-0.04% BSA + [M] 1 millimolar (mM) DTT + 0.2 U/µL RNase Inhibitor).

20 Check that cells are fixed using trypan blue (90 µL of trypan blue with 10 µL of cells).

21 Count cells on a disposable c-chip hemocytometer contained in a petri dish, and resuspend cells to a final density of about 2000 cells/µl in the SSC cocktail.

22 Proceed to standard scRNAseq protocol.

Additional Procedures: Basic Biosafety Cabinet Setup

23

Biosafety cabinets must be minimally equipped with:

- Overlapping plastic-backed underpads to cover work surface of biological safety cabinet, spritz with disinfectant.
- One container of Vesphene® III disinfectant, with Kimtowel inside the container.
- If pipettes are used, a metal petri dish can (labeled with date and name) containing a bag with a layer of disinfectant inside of it.
- Plastic roller bottle or other plastic capped container, half filled with disinfectant.

- Biohazard waste bag labeled with name and date, securely attached to inside of biological safety cabinet with tape.
- Large waste bucket with biohazard bag. The lid of the bucket must be labeled with name and date.
- Several rubber bands.
- A 500 ml squirt bottle with disinfectant, markers, pipetmen, and pipette aid.
- Add equipment and other supplies as needed.

Additional Procedures: Standard item removal procedure

24

Note

This is the standard procedure for the removal of any item from a biological safety cabinet. Anything opened in the biological safety cabinet must not be opened outside of the cabinet. This includes pipette boxes, rotors, and rotor adaptors. A pipetmen or any pipetting device must only be removed from a biological safety cabinet in a secondary container, such as a Rubbermaid box with a lid (preferably one with a gasket). The container is removed from the biological safety cabinet as described **Basic Biosafety Cabinet Setup**.

The following applies to the removal of items such as: metal cans, pipet boxes, any secondary container, sealed centrifuge buckets or rotors, and the electroporation chamber unit.

24.1 The item is wiped down with disinfectant.

24.2 Wipe the outer pair of your gloves with disinfectant, remove gloves, and throw them into red waste bag in the biological safety cabinet.

24.3 Gloved hands can then be removed from the cabinet; a fresh pair of outer gloves is put on and you then re-enter the biological safety cabinet.

24.4 Wipe down gloves and the surface of the item with disinfectant, then remove item from the cabinet.

25 For vortexers and other small equipment:

25.1 Prior to use, the equipment is placed into the biohazard bag with the cord sticking out of the opening of the bag. Using a twist tie or rubber band to secure the opening around the cord, and place the unit in the biological safety cabinet. If using a vortexer, reinforce the area of the bag over the cup with two pieces of tape.

25.2 To remove the equipment, wipe down the area around the opening of the bag and the cord. Wipe down the outer gloves and remove.

25.3 Remove hands from the biological safety cabinet, don a fresh pair of outer gloves and re-enter the cabinet.

25.4 Wipe hands again and remove the twist tie or rubber band. Keeping one hand on the bag, reach into the bag with the other hand and remove the equipment from the bag and the biological safety cabinet in one movement. Throw bag into waste bag inside of the biological safety cabinet.

26 For tube racks:

1. Racks are placed into a waste bag, which is folded over itself. The bag is removed from the biological safety cabinet with the standard item removal procedure into another bag and closed with a rubber band.
2. The outer bag should be labeled “SAVE”. It is then ready for the autoclave.

Additional Procedures: Pipetting and other liquid transfers

27

- Use mechanical pipetting devices only. NO MOUTH PIPETTING is permitted.
- Do not forcefully eject liquid from pipettes and avoid expelling the last drop from the pipette.
- When dispensing the liquid from a pipette, the tip should be below the fluid level or as close as possible to the agar level. Splatter from dropping liquid will create aerosols.
- Aerosol barrier tips should be used with pipetters.
- Re-suspension of viable material must be done so the tip is below the liquid level.
- The transfer of infectious liquids must be done in ways that reduce the generation of aerosols.
- Transfers using pipettes or pipetmen should always be done with the tip of the pipette or pipette tip positioned below the top of the liquid as it is flowing into the receptacle. Do not blow out the last drop.
- Transfer of cultures from culture vessels into tubes should always be done with pipettes and should never be poured. Washes may be poured off into a waste container in the biological safety cabinet.
- Aerosol barrier tips should be used with pipettes.
- No glass pipettes should be used, with the exception of 6-inch Pasteur pipettes, which are only used to remove liquid from electroporation cuvettes. Waste Pasteur pipettes are disposed of in a sharps container.
- After transfer with a pipette or pipette tip, pull up a quantity of disinfectant into pipette or tip and then flush out. Eject tip into waste container in the biological safety cabinet or place the pipette into the waste container in the biological safety cabinet.

Additional Procedures: Centrifugation

28 10m

Note

All low-speed centrifugation must be done in capped tubes in centrifuge safety cups or centrifuge rotors that provide a gasket for containment AND can be removed to the biological safety cabinet for loading and unloading without opening.

The following procedures must be followed during centrifugation.

- Only use appropriate tubes/bottles and check maximum centrifuging speed.
- Before centrifuging, inspect tubes/bottles for cracks and stress marks.
- Make sure the correct adapters are in place.
- Fill and decant all centrifuge tubes/bottles within the biological safety cabinet. Wipe outside with disinfectant before placing into the rotor.
- If decontamination of a rotor or bucket is required, soak rotor / buckets and lids in a container of disinfectant for 00:10:00 in the biological safety cabinet. Rinse with water. Then wipe down, replace cover, and remove from the biological safety cabinet with the standard procedures.

Additional Procedures: Tabletop Centrifugation - Swinging Bucket

29 Centrifugation in the tabletop centrifuge is always done with the aerosol proof buckets with gasketed caps. The sealed buckets are never opened outside of the biological safety cabinet, regardless of whether or not they contain tubes.

30 Take the buckets or rotors into the biological safety cabinet and open it.

30.1 Wipe down the tubes with disinfectant and place into the buckets.

30.2 Replace the caps and wipe down the buckets with disinfectant.

30.3 Remove the buckets from the biological safety cabinet using the standard removal procedure.

31 After centrifugation, take the buckets to the biological safety cabinet and remove the lids. Remove the tubes from the buckets and wipe down the inside of the lids with disinfectant and secure onto the buckets.

32 Generally, the buckets do not have their interiors wiped out unless there is a small amount of leakage. In that case, the buckets are thoroughly wiped out with disinfectant and resealed.

33 It is very important that users check gasket integrity each time the buckets are used.

34 In case of tube breakage, let the bucket rest in the centrifuge for 00:10:00, carefully remove the bucket and inspect the seal. 10m

34.1 If it is compromised, consider the tube failure an “out of the cabinet” spill and proceed accordingly.

34.2 If the gasket is intact, remove the bucket to the biological safety cabinet and clean out tube debris.

35 Soak the buckets and lids in a container of disinfectant for 00:10:00 in the biological safety cabinet. Rinse with water. 10m

36 Then wipe down, replace the covers, and remove from the biological safety cabinet with the standard procedure. Return the bucket to the centrifuge.

Additional Procedures: Tabletop Centrifugation - Micro/High Speed

37 Remove rotor and place into biological safety cabinet.

38 Remove aerosol cover. Inspect gasket, replace if necessary.

39 Wipe down tubes with disinfectant. Place tubes in rotor and replace cover.

40 Remove rotor from biological safety cabinet with standard removal procedure.

41 Place into centrifuge, commence run. After run, remove rotor and place into biological safety cabinet.

42 Remove tubes. Wipe down interior of rotor with disinfectant. Check gasket. Remove rotor from biological safety cabinet with standard procedure. Return rotor to centrifuge.

43 In case of tube breakage during run: Let rotor rest in centrifuge for 00:10:00 . 10m

43.1 If you open the centrifuge and it looks like a tube leaked, immediately close the lid and consider this an out of the biological safety cabinet spill. Proceed accordingly.

43.2 If there is no leakage, then remove rotor, and keeping it level; remove it to the biological safety cabinet.

44 In the biological safety cabinet, take off the cover of the rotor and remove all tubes and debris. Soak rotor and cover in a container of disinfectant for 00:10:00 in the biological safety cabinet. 10m

45 Rinse with water. Then wipe down, replace the cover, and remove.

Inactivated SARS-Cov-2 TCID determination

46

> **Note**
>
> Notes:
>
> - Test each sample in triplicate
> - Virus stock will be used as a positive control.

Seed Vero E6 cells in complete media (EMEM/10% FBS/1% Pen/Strep) into 96-well microtiter plates (8 x 10^3cells/well) or 18-48 h before the assay in a BSL2 lab. Optimal cell density is around 2x10^4 cells/cm^2.

47 On the day of assay, transfer the Vero E6 cells to the CO_2 incubator in the BSL3 lab in a secondary container.

48 Prepare 12 x 5-fold serial dilutions. The first dilution should be a 1:1 mixture of warm infection medium (EMEM/3%FBS) and the 2000 cell/ul in SSC cocktail.

49 Transfer 50 µL of virus dilution per well onto the Vero E6 cells.

49.1 Wipe the outside of plate with disinfectant and place into a plastic secondary container with lid.

49.2 Take the secondary container with the culture plate from the biosafety cabinet according to the protocol "Standard Item Removal Procedure", and place into the CO_2 incubator.

49.3 Carefully open one corner of the lid to allow air exchange.

49.4 Incubate for 01:00:00 at 37 °C. 1h

50 After 1 h viral adsorption, transfer the secondary container with the culture plate to BSC, remove plate, remove media, and add 150 µL of warm post infection medium at 37 °C to each well.

50.1 Wipe the outside of plate with disinfectant and place into a plastic secondary container with lid.

50.2 Take the secondary container with the culture plate from the biosafety cabinet according to the protocol "Standard Item Removal Procedure" and place into the CO2 incubator.

50.3 Carefully open one corner of the lid to allow air exchange.

51 Incubate cultures at 37 °C with 5% CO_2 for up to 6 days, until CPE is evident.

51.1 To monitor cultures for CPE, place a piece of absorbent bench coat paper under a microscope.

51.2 Transfer the secondary container with the culture plate from the CO_2 incubator to bench, on top of the paper, ensuring that the lid is secured to the secondary container before removing it from the incubator.

51.3 Once on the bench, look through the side of the container to see if any liquid has spilled out of the tray.

- If there is liquid, move the container to the biosafety cabinet and proceed to decontaminate the tray, and secondary container.
- If there is no spilled liquid, carefully remove the lid to the secondary container and place the tissue culture plate onto the microscope stage.

Note

Do not remove the lid from the tissue culture plate.

51.4 After examining the plate under the microscope, replace the tray back into the secondary container, secure the lid and return the container to the CO_2 incubator, and then carefully open one corner of the lid to the secondary container for air exchange.

52 Score positive and negative wells based on CPE observed and calculate TCID50/ml using the Spearman-Karber method. Analyze and validate viral inactivation.
